# The cross-talk of lung and heart complications in COVID-19: Endothelial cells dysfunction, thrombosis, and treatment

**DOI:** 10.3389/fcvm.2022.957006

**Published:** 2022-08-05

**Authors:** Langjiao Liu, Haijiao Jing, Xiaoming Wu, Mengqi Xiang, Valerie A. Novakovic, Shuye Wang, Jialan Shi

**Affiliations:** ^1^Department of Hematology, First Affiliated Hospital of Harbin Medical University, Harbin Medical University, Harbin, China; ^2^Department of Research, VA Boston Healthcare System, Harvard Medical School, Boston, MA, United States; ^3^Department of Medical Oncology, Dana-Farber Cancer Institute, Harvard Medical School, Boston, MA, United States

**Keywords:** COVID-19, cardiopulmonary, phosphatidylserine, microparticles, endothelial cells, antithrombotic, sequelae

## Abstract

The pandemic respiratory illness SARS-CoV-2 has increasingly been shown to be a systemic disease that can also have profound impacts on the cardiovascular system. Although associated cardiopulmonary sequelae can persist after infection, the link between viral infection and these complications remains unclear. There is now a recognized link between endothelial cell dysfunction and thrombosis. Its role in stimulating platelet activation and thrombotic inflammation has been widely reported. However, the procoagulant role of microparticles (MPs) in COVID-19 seems to have been neglected. As membrane vesicles released after cell injury or apoptosis, MPs exert procoagulant activity mainly by exposing phosphatidylserine (PS) on their lipid membranes. It can provide a catalytic surface for the assembly of the prothrombinase complex. Therefore, inhibiting PS externalization is a potential therapeutic strategy. In this paper, we describe the pathophysiological mechanism by which SARS-CoV-2 induces lung and heart complications through injury of endothelial cells, emphasizing the procoagulant effect of MPs and PS, and demonstrate the importance of early antithrombotic therapy. In addition, we will detail the mechanisms underlying hypoxia, another serious pulmonary complication related to SARS-CoV-2-induced endothelial cells injury and discuss the use of oxygen therapy. In the case of SARS-CoV-2 infection, virus invades endothelial cells through direct infection, hypoxia, imbalance of the RAAS, and cytokine storm. These factors cause endothelial cells to release MPs, form MPs storm, and eventually lead to thrombosis. This, in turn, accelerates hypoxia and cytokine storms, forming a positive feedback loop. Given the important role of thrombosis in the disease, early antithrombotic therapy is an important tool for COVID-19. It may maintain normal blood circulation, accelerating the clearance of viruses, waning the formation of MPs storm, and avoiding disease progression.

## Introduction

SARS-CoV-2 was initially thought to be a disease that primarily affected the lungs, but as data accumulated, it emerged that it could also affect other organs and cause multiple organ dysfunction. The most prominent extrapulmonary complication is cardiovascular disease (1). And its occurrence is often closely associated with lung disease (2). While in some patients, the virus may directly infect the target organ through the angiotensin-converting-enzyme 2 (ACE2) receptor, resulting in extrapulmonary complications, in most patients, SARS-CoV-2 first invades the lungs and then progresses to more severe multi-organ failure. Because many patients show only respiratory symptoms (fever, cough, etc.) in the initial stage of infection (3). Thrombosis plays an integral role in pulmonary and cardiovascular complications (4). Ackermann et al. reported that histologic analysis of COVID-19 patients showed widespread thrombosis with microangiopathy, and that the prevalence of microthrombi in COVID-19 patients was nine times higher than in flu patients (5). The same was true for cardiovascular disease. In the laboratory, myocardial injury is associated with coagulation indexes such as elevated D-dimer (6). An experimental study also indicated that myocardial fibrin microthrombosis was common in patients with COVID-19 (7).

Dysfunction of endothelial cells is considered to be the main factor for thrombosis (8). Under normal conditions, endothelial cells play an anticoagulant role through anti-platelet adhesion, synthesis of antithrombin and other coagulation factors, promotion of fibrinolysis and barrier protection. Viral infection causes endothelial cell damage, resulting in disorders of the coagulation and fibrinolytic systems, ultimately leading to thrombosis (9). However, we found that MPs were rarely mentioned in COVID-19 studies. MPs are membrane vesicles released when endothelial cells are activated, damaged or undergoing apoptosis (10). MPs participate in inflammation and coagulation through cellular components such as proteins, lipids, nucleic acids and PS (11–13). The PS exposed on the surface of MPs provides binding sites for endogenous and exogenous FXase complexes and the prothrombin complex, provides a platform for the coagulation cascade, thereby promoting clot formation. Our team has shown in previous studies that when endothelial cells are damaged, PS exposure results leads to abnormalities in the blood coagulation system (14, 15). Therefore, understanding the physiology of endothelial cells injury and the pro-coagulant activity of PS externalization could provide a new prospect for the treatment of COVID-19.

Before there were no specific drugs, antithrombotic therapy for COVID-19 reached a consensus (16). Many studies have also shown that thrombotic-related sequelae (such as pulmonary fibrosis, chest pain, etc.) can occur in COVID-19 patients during convalescence (17, 18). The normal function of blood circulation is to provide oxygen and nutrients to the body and to remove metabolites. After thrombosis, blood vessel blockage hampers the clearance of metabolites and viruses in a timely and efficient manner, aggravating the progression of the disease. Therefore, early antithrombotic therapy is especially important. Unobstructed blood circulation speeds recovery by facilitating the removal of viruses and cytokines, preventing COVID-19 patients from developing serious complications and therefore reducing mortality. Rentsch's study has shown that early initiation of prophylactic anticoagulation reduced 30-day mortality with no increased risk of serious bleeding (19), supporting our opinion.

## Cardiopulmonary complications of COVID-19 and their interactions

### Pulmonary complications

Lungs are the primary target of SARS-CoV-2 invasion. Most people with mild symptoms have fever, cough and difficulty breathing, but a small number of patients, 5–10%, will develop severe respiratory failure and acute respiratory distress syndrome (ARDS) (20–22). According to relevant literature, the mortality rate of ARDS in COVID-19, at 26–61.5%, is higher than that of ARDS secondary to other diseases (23). Many studies suggest that pulmonary embolism is also very common in COVID-19 patients. Poissy et al. showed that pulmonary embolism (PE) incidence was 20.6% in COVID-19 patients in 1 month in 2020, which was significantly higher than the incidence of PE for other causes of admission during the same period (24). More frightening, many data confirm that some patients still have long-term pulmonary sequelae after discharge from the hospital, including some patients diagnosed with only mild acute phase disease (25). A cohort study by Mo et al. noted that impaired diffusion ability was the most common abnormal lung function among discharged COVID-19 survivors (47.2%) (26). Frija-masson et al. reported an isolated reduction in lung diffusion capacity in 13 of 50 patients (26%) (27). In addition, Cueto-Robledo et al. found severe pulmonary hypertension in patients discharged from hospital (28). The pathogenesis of long COVID-19 is still unknown. Persistent viral shedding (29), inflammatory environment (17), and coagulation issues may be involved in sequelae-related pathologic processes (30).

### Cardiac complications

Cardiovascular complications in COVID-19 occur at a high rate and tend to have a poor prognosis. Observational studies have shown that about 5.3% of patients with COVID-19 developed new-diagnosed secondary acute myocardial infarction (AMI) (31). In addition, related reports have shown that 33.3–39.3% were diagnosed with non-obstructive coronary artery disease (32, 33). This suggests that COVID-19 itself may be associated with endothelial dysfunction and hypercoagulability (34). Arrhythmias are a common manifestation of COVID-19 cardiovascular complications. In a study of 138 patients in China, new atrial fibrillation, heart block and ventricular arrhythmias are common in COVID-19, with an incidence of 44.4% in 36 intensive care unit (ICU) patients (35). Arrhythmia is caused by multiple factors. In addition to drugs (such as hydroxychloroquine) and inflammation (36), it can also be secondary to myocardial injury. According to the survey, malignant arrhythmias are more frequent in patients with elevated TnT levels during hospitalization (37). An increased risk of clinical acute myocardial ischemia was also associated with COVID-19. In a large, controlled trial, the incidence of acute myocardial ischemia after COVID-19 diagnosis was five times higher than in the control group (38). Heart failure, myocarditis, and cardiogenic shock have also been reported (39–41). There are also many reports about the sequelae associated with cardiovascular thrombosis. Tschöpe et al. and Fan et al. reported on several cases of coronary angiography of patients discharged from hospital with COVID-19, including left anterior descending artery obstruction in one patient and left anterior descending artery obstruction (in 25% of participants) (42, 43). Ayoubkhani and others also reported a composite outcome (major adverse cardiovascular event), including heart failure, myocardial infarction, stroke and arrhythmia (44).

### Lung-heart interplay

**Figure 1:**
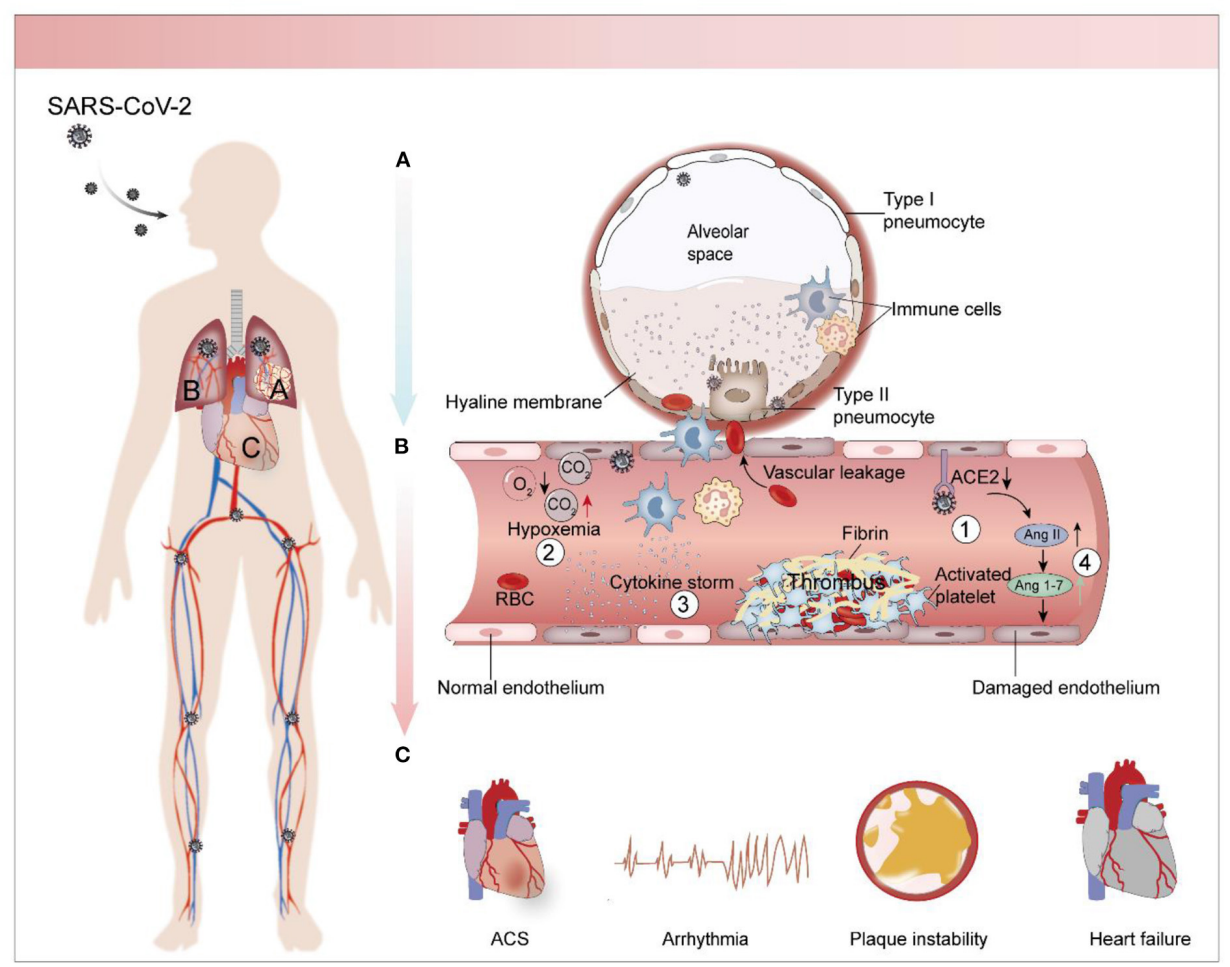
Pathological mechanism of cardiopulmonary complications caused by SARS-CoV-2. **(A)** Pathophysiology of SARS-CoV-2-infected lungs. The virus infects alveolar type II epithelial cells and pulmonary capillary endothelial cells through the respiratory tract and stimulates immune cells to release inflammatory factors, causing cytokine storm. As viral replication enters into the bloodstream, it stimulates endothelial cell shrinkage and narrows the pulmonary capillary lumen. This causes a local lack of oxygen to the lungs. Endothelial cells damage worsens as the disease progresses, stimulating clotting cascades in the pulmonary capillaries, which leads to further increases in capillary pressure and resulting in pulmonary hypertension. This causes the release of plasma, along with inflammatory cells and coagulation factors, into the alveolar cavities. Evaporation concentrates these substances and forms a peptone-like translucent film in the alveolar cavity. **(B)** Endothelial cells injury and thrombosis. Endothelial cells injury caused by SARS-CoV-2 is multifactorial. Some viruses infect endothelial cells through ACE2 receptor, and indirect mechanisms including hypoxia, RAAS imbalance and cytokine storm also aggravate endothelial cells injury. The injured endothelial cells stimulate the coagulation cascade and promote the formation of thrombosis. **(C)** Cardiac complications associated with COVID-19. Including arrhythmias, heart failure, coronary syndrome, which are often closely associated with thrombosis.

Pulmonary and cardiovascular complications in COVID-19 are often correlated. Heart and lung are inseparable organs in that they relate to the two major circulation pathways of the human body: systemic circulation and pulmonary circulation. As lung disease progresses, it can cause heart stress and vice versa. Mancini et al. found that circulatory damage caused by changes in long COVID related cardiac function also included reduced pulmonary perfusion (suggesting that it might be related to dyspnea), which in turn resulted in reduced cardiac output and further burdened the heart (45). Several other studies have identified right ventricular dysfunction and diastolic dysfunction as being a result of fibrous lung injury, pulmonary hypertension, and clot burden in critically ill patients (46–48). Guo et al. found that ARDS occurred more frequently in COVID-19 patients with myocardial injury [30 [57.7%] vs. 16 [11.9%]] (49). This evidence confirms the heart-lung connection. As previously mentioned, thrombosis is a major factor in cardiopulmonary complications in COVID-19 patients with endothelial cells injury being a common mechanism bridging the two systems. Von Willebrand Factor (vWF), a major marker of endothelial cell dysfunction, was found to be significantly elevated in laboratory examination of COVID-19 patients, and its value was positively correlated with the severity of the disease (50, 51). The virus invades the lung through the respiratory tract and mainly infects alveolar type II epithelial cells, causing immune cells to release inflammatory mediators and form cytokine storms, resulting in local hypoxia (52). At the same time, some of the virus escapes the phagocytosis of immune cells and further invades the adjacent pulmonary endothelial cells (Figure 1A). Under the direct invasion of virus, hypoxia, imbalance of Renin-angiotensin-aldosterone system (RAAS) and cytokine storm, the integrity and permeability of endothelial cells are damaged (Figure 1B). Damaged endothelial barrier combined with cytokine storm can promote the formation of acute endodermatitis in the lung and extend into systemic circulation, resulting in intravascular blockage and thrombosis that may lead to myocardial injury, ischemic heart disease and other diseases (Figure 1C). The blockage of the blood vessels in turn increases dead-space ventilation, further exacerbating hypoxia, creating a vicious cycle (53).

## The reasons for endothelial cells dysfunction in COVID-19

The main pathophysiological mechanisms of SARS-CoV-2 injury to endothelial cells include direct mechanisms (direct viral invasion) and indirect mechanisms (hypoxia, RASS imbalance, cytokine storm).

### Direct virus infection

The entry of SARS-CoV-2 into cells is mediated by the recognition of the host cell ACE2 receptor, which requires the mediation of transmembrane protease receptor serinase 2 (TMPRSS2) (54). ACE2 receptors are expressed in multiple organs, such as the lung, heart, kidney, intestine, etc., primarily on endothelial cells (55, 56). Varga's team found viral particles in the renal endothelial cells of COVID-19 patients and noted that direct viral infection through the endothelium may lead to extensive endothelial dysfunction and apoptosis (57). Similarly, Ackermann et al. examining autopsies of lungs from patients who died of COVID-19 demonstrated that viral infection increases endothelial ACE2 expression (5). However, it remains controversial whether SARS-CoV-2 directly infects endothelial cells through ACE2 receptor. Some studies have argued against this view, suggesting that endothelial ACE2 has a low basal expression level and may escape SARS-CoV-2-mediated cell entry (58). Yet there's also support for the presence of ACE2 receptors on endothelial cells. In one study, Ahmetaj-Shala et al. reported detectable levels of ACE2 transcripts in human endothelial cells by reanalyzing the human tissue transcriptome database (59). A representative study by Lei et al. showed that the S protein of SARS-CoV-2 could damage vascular endothelial cells by down-regulating ACE2 (60). Qin et al. demonstrated SARS-CoV-2 in capillary endothelial cells using RNA-seq analysis in a mouse model of COVID-19 (61). A single-cell RNA-seq analysis of non-human primates also showed increased expression of SARS-CoV-2 receptor ACE2 in alveolar epithelial barriers, cardiomyocytes, and vascular endothelial cells (62). Therefore, the role of direct viral infection in endothelial cell injury needs to be further studied.

### Hypoxemia

In the early stage of COVID-19, a small number of SARS-CoV-2 viruses infect nasal cilia cells and secretory cells through the air, activating the innate immune response. Further some active viruses escape the clearance of immune cells and enter the alveolar cavity through the trachea. The virus then infects the type II epithelial cells of the alveoli and the adjacent endothelial cells of the air-blood barrier. This process causes alveolar space damage, recruiting a variety of immune cells which release inflammatory cytokines and cause a cytokine storm (63). Alveolar capillary endothelial cell injury and cytokine storm activate the coagulation cascade to form pulmonary microthrombosis, resulting in an imbalance of pulmonary ventilation and blood flow ratio, leading to local pulmonary hypoxia (64). When viral replication reaches the bloodstream, it stimulates endothelial cell shrinkage, narrowing the pulmonary capillary lumen. This increases pressure in the capillary network, eventually resulting in pulmonary hypertension, which forces plasma into the alveolar cavities along with inflammatory cells and blood coagulation factors. As some of the water in the alveolar cavity evaporates, these substances form a peptone-like translucent film in the alveolar cavity, severely impairing the function of the gas and blood barrier (65). This causes blood with low oxygen saturation to move from the capillary network of the alveolar wall into systemic circulation and causing serious hypoxia.

### Imbalance of RAAS

RAAS is a pressure-boosting regulation network produced by the kidneys, causing vascular smooth muscle contraction, water and sodium retention and resulting in pressure-boosting effects. Under normal circumstances, ACE2 promotes the degradation of Ang II and maintains equilibrium (66). However, due to ACE2 depletion in COVID-19 patients, AngII levels are elevated, causing vasoconstriction and enhanced vascular permeability, thereby damaging endothelial cells (67).

### Cytokine storm

Cytokine storm can not only promote the formation of hypoxia, but also directly cause endothelial injury (68). Wu et al. confirmed that IL-1β and TNF-α (TH17 and TH1 cells express) promote TH17 response. They increase endothelial permeability and vascular leakage (69). High levels of TNF-α and IL-1β may also down-regulate the expression of Kluber-like factor 2 (KLF2) in human endothelial cells, leading to monocyte adhesion and endodermatitis (70). In addition, activated neutrophils have been shown to release neutrophil extracellular traps (Nets) in COVID-19 patients. In an experimental study of COVID-19, Nets production was positively associated with disease severity and mortality. Originally produced to kill pathogens, Nets can also activate endothelial cells when produced in excess (71). Meanwhile, Laforge et al. pointed out that in COVID-19, neutrophils produce excessive ROS, which intensifies the host's immunopathological response, as well as tissue damage, leading to more severe disease (72).

## Endothelial cells damaged in COVID-19 leads to thrombosis

After the activation or injury of endothelial cells, the most serious consequence is the formation of thrombi. First, endothelial cells are damaged and shed, exposing collagen, activating platelets and promoting their adhesion and aggregation through vWF released by endothelial cells, forming platelet thrombosis. At the same time, MPs released from damaged endothelial cells participate in the clotting response by exposing PS on their surfaces, promoting fibrinogen production and further consolidating platelet thrombosis. In addition to procoagulant factors, damaged endothelial cells can secrete fibrinogen activator inhibitor 1 (PAI-1), which inhibits fibrinolysis (73). Thrombin can also induce activation of endothelial cells through protease activated receptor 1 (PAR1) (74), which in turn aggravates the injury of endothelial cells.

**Figure 2:**
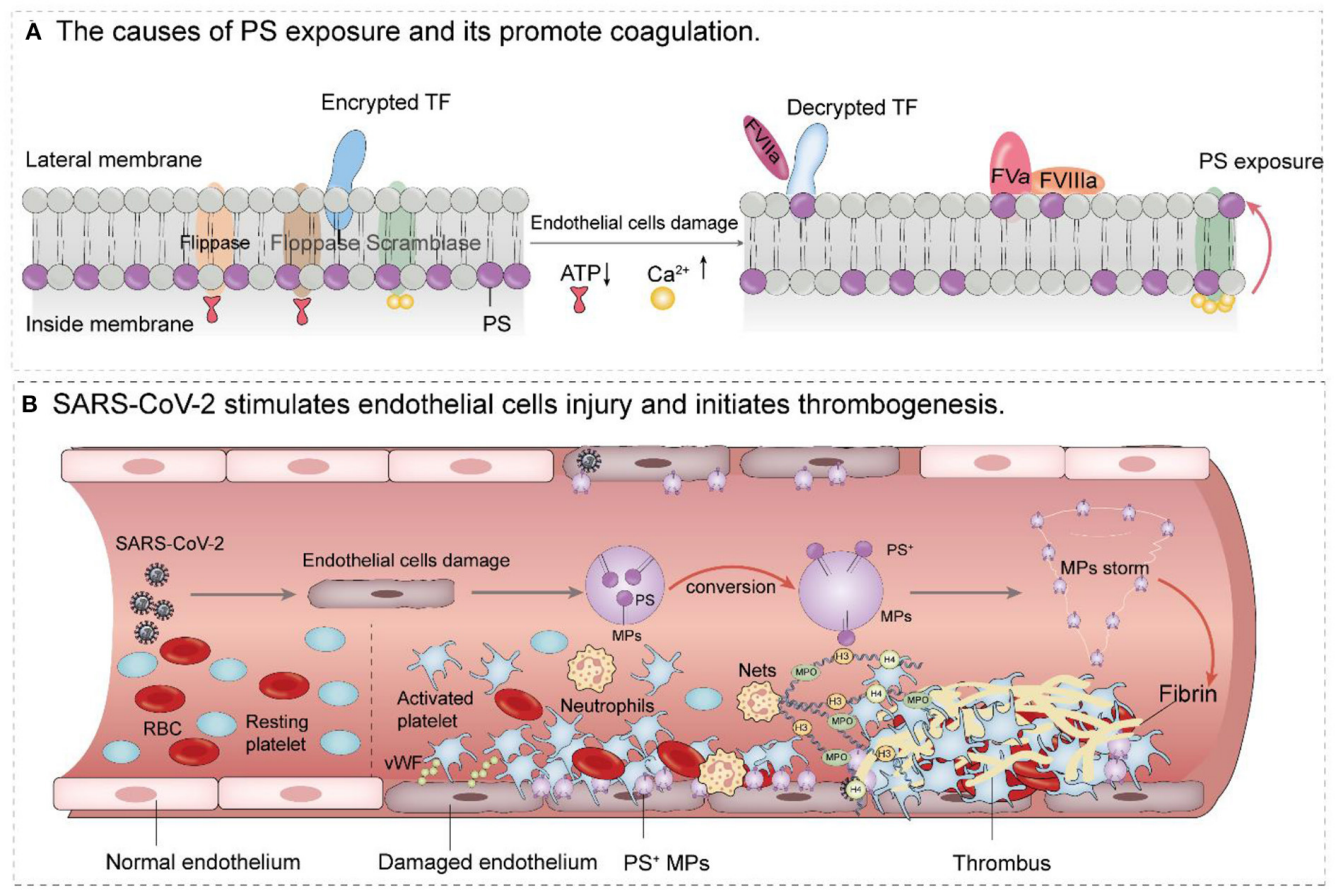
Mechanism of PS^+^ MPs promoting thrombosis after endothelial cells injury. **(A)** When endothelial cells are damaged, the two ATP-dependent enzymes are unable to function due to energy depletion. However, as intracellular Ca^2+^ concentration, the activated scramblase flips PS to the outer membrane where it initiates coagulation PS provides a scaffold for the activity of factors VIII and V, which binding membranes through their C2 domains, and factors II, VII, IX, and X, which bind using their Gla domains. These factors combine to form the intrinsic (FXIa-FVIIIa-Ca^2+^-PL) factor X enzyme complex and the prothrombin complex (FXa-FVa-Ca^2+^-PL) Damaged endothelial cells can also upregulate the expression of tissue factor, which is activated in the presence of membrane PS and then forms the TF-VIIa complex that participates in exogenous coagulation. **(B)** SARS-CoV-2 causes endothelial dysfunction and thrombosis. After endothelial cell injury, stimulated platelets were transformed from the resting state to the activated state, and PS^+^ microparticles were released from the surface of endothelial cells to form a particle storm. Platelet adhesion, coupled with PS exposure, stimulates the activation of the clotting cascade, which ultimately leads to thrombosis (The involvement of neutrophils and Nets in endothelial cell injury and thrombosis is also shown in **B**).

PS is a glycerolipid that makes up part of the cell membrane. Under normal conditions, PS is mainly distributed in the inner layer of the plasma membrane. This asymmetric distribution is maintained through the action of three enzymes: ATP-dependent flippase and floppase, and ATP-independent but Ca^2+^ dependent scramblase. When endothelial cells are damaged, the two ATP-dependent enzymes are unable to function due to energy depletion, but intracellular Ca^2+^ concentration increases. The activated scramblase flips PS to the outer membrane where it initiates coagulation (75). The specific mechanism is as follows: on the one hand, PS provides a scaffold for the activity of factors VIII and V, which binding membranes through their C2 domains, and factors II, VII, IX, and X, which bind using their Gla domains. These factors combine to form the intrinsic (FXIa-FVIIIa-Ca^2+^-PL) factor X enzyme complex and the prothrombin complex (FXa-FVa-Ca^2+^-PL) (76–80). On the other hand, damaged endothelial cells can upregulate the expression of tissue factor, which is activated in the presence of membrane PS and then forms the TF-VIIa-Ca^2+^-PL complex that participates in exogenous coagulation (81). The coagulation cascade leads to the conversion of prothrombin to thrombin which then catalyzes the formation of a fibrin network (Figure 2A). In this way, fibrin and platelet thrombosis form a stronger thrombus. With endothelial cell damage in severe COVID-19 patients, a large number of PS^+^ MPs are released, forming an MPs storm and ultimately causing serious thrombotic events (Figure 2B).

## Treatment

In conclusion, whether in patients with pre-existing comorbidities or in previously healthy patients, the heart and lung complications in COVID-19 are essentially caused by thrombosis. We have mentioned that the virus causes the endothelial cells destruction which leads to thrombosis. In turn, thrombosis exacerbates the vicious cycle of hypoxia and endothelial damage which eventually aggravates the progression of the disease. Therefore, in the absence of specific drugs for the elimination of SARS-CoV-2, in addition to conventional treatments such as antiviral drugs, attention should be paid to antithrombotic therapies, oxygenation and preventing endothelial cells damage.

### Early antithrombotic

The therapeutic benefits of anticoagulation in COVID-19 are widely accepted. Low molecular weight heparin (LWMN) is currently the most commonly used anticoagulant for COVID-19. Heparin is recommended because aside from its powerful blood-thinning properties, it also has anti-inflammatory and antiviral effects, and can be injected subcutaneously to reduce the adverse reactions of the drug (82) (a study has confirmed that SARS-CoV-2 spike protein S1 subunit contains a receptor binding domain structure that can interact with heparin) (83). Meanwhile, inhibition of platelet activation has also been reported to have beneficial effects. A recent study demonstrated that in-hospital aspirin use was associated with a lower incidence of in-hospital death (84). Antiplatelet drugs include the thrombotic inhibitor aspirin, P2Y12 inhibitor clopidogrel, phosphodiester RAASe inhibitor dipyridamole, among others (85). Aspirin is the drug of choice for the prevention of arterial thrombosis in high-risk patients with cardiovascular risk factors (86). Thrombolytic therapy is more controversial. In one study however, the use of tissue plasminogen activator (tPA) improved oxygenation in patients with severe COVID-19 (87). Therefore, more exploration of the use of thrombolytic therapy in severely ill patients is warranted.

However, the timing of anticoagulant therapy remains controversial. A randomized controlled trial (RCT) showed that therapeutic anticoagulant therapy did not reduce mortality in severe COVID-19 patients (88). Similarly, another study showed that in patients with severe COVID-19, treatment with antiplatelet drugs was less likely to improve the number of days without organ support over 21 days than treatment without antiplatelet drugs (89). In contrast, in another report of 465 patients with moderate COVID-19, therapeutic heparin reduced mortality at 28 days (23 patients with therapeutic heparin (10.1%) vs. 38 patients (16.0%) given prophylactic heparin). And the risk of major bleeding seems low [Two (0.9%) patients received therapeutic heparin and four (1.7%) patients received prophylactic heparin] (90). Another retrospective analysis of 413 patients showed that early anticoagulant therapy with relatively high doses of LMWH improved clinical outcomes and shortened hospital stays for COVID-19 patients (91). Gonzalez-ochoa et al. reported that early use of Sulodexide improved clinical outcomes. Compared with the control group, sulodexide had a lower hospitalization rate (17.7 vs. 29.4% [*P* = 0.03]) and fewer patients requiring supplemental oxygen therapy (30 vs. 42% [*P* = 0.05]) (30% vs. 42% [*P* = 0.05]). After 2 weeks, d-dimer levels >500 ng/dL were reduced (22 vs. 47% [*P* < 0.01]) and there was no difference in thromboembolic events, major bleeding, and mortality (92). The above results highlight the necessity of early antithrombotic therapy. In fact, although the symptoms of early-stage patients are mild, and D-dimer is normal or only slightly increased, it is still possible that fibrinolytic activity is present and that the body is in the early stages of coagulation activation. Early endothelial cells damage and PS externalization may be too subtle to be detectible at this stage. Our main task is to prevent further externalization of PS to prevent further clotting response. Therefore, early antithrombotic therapy is necessary. It guarantees the alveolar has effective blood perfusion, avoids endothelial to activation, protects the air/blood exchange, ensures unobstructed pulmonary circulation, and maintains saturated blood oxygen levels throughout the systemic circulation, which prevents endothelial cells damage and thrombosis. In patients with advanced stage disease, there is already severe thrombosis in the body, thus cytokines and viruses cannot be cleared in time, further aggravating the difficulty of treatment.

### Other drugs

#### Vaccination

Vaccination is well-known to lessen the severity of acute phase COVID-19, and some reports also indicate that Long-COVID symptoms are less prevalent and milder in vaccinated people. A case-control study involving 1.2 million people who received two doses of vaccine had a lower chance of having symptoms lasting 28 days or longer (odds ratio 0.51 [95% CI 0.32–0.82]; *P* = 0.0060) (93). However, long-term sequelae are still found in some vaccinated patients (94). The main reason why vaccination is ineffective for some patients is that the pathogenesis of COVID-19 and Long COVID is multifactorial. Persistent viral infection and thrombosis are the main factors. Therefore, we still advocate early antithrombotic therapy in vaccinated patients.

### Oxygen therapy

Hypoxemia induced by COVID-19 plays an important role in the process of endothelial cells damage, such that the improvement of blood oxygen saturation can also improve the course of the disease. A controlled trial showed that high-flow oxygen significantly reduces the need for mechanical ventilation support in severe COVID-19. Compared with those receiving conventional oxygen therapy, the incidence of endotracheal intubation was reduced (34.3 vs. 51.0 %) and the median 28-day clinical recovery time was shorter (11 vs. 14 days) in patients receiving high-flow oxygen therapy (95).

### Lactadherin

Our group has previously demonstrated that lactadherin can specifically bind to PS. Because lactadherin contains a C2 domain homologous to those on some clotting factors, it can act both as a probe and a competitive inhibitor to factor V and VIII binding. The structure of lactadherin also includes an RGD domain that has been shown to act as an “eat me” signal that could aid in the clearance of PS-exposing damaged or apoptotic cells. Although there are few reports on the application of lactadherin in COVID-19, the procoagulant effect of PS in COVID-19 cannot be ignored. Therefore, lactadherin is a promising agent to study in terms of inhibiting the effects of PS externalization (79, 80, 96).

### Statins

In previous influenza studies, statins have been shown to reduce endothelial cell activation, inhibit procoagulant pathways, and enhance antithrombotic effects. A retrospective study has shown that in-hospital statin use is associated with a reduced risk of death among COVID-19 patients. In this article, Cox models were used to find that the 28-day all-cause mortality was 5.2% in the statin group and 9.4% in the non-statin group, with an adjusted hazard ratio of 0.58 (97).

### ACE2, TMPRSS2 inhibitors

ACE2 and TMPRSS2, as important vectors of SARS-CoV-2, are potential therapeutic targets. Damir Bojadzic et al. demonstrated that several small molecule inhibitors targeting the SARS-CoV-2 spike protein and human ACE2 (hACE2) prevented two different SARS-CoV-2-S-expressing pseudoviruses from entering hACE2-expressing cells (98). In a mouse model, TMPRSS2 inhibitor (nafamostat) protected mice against SARS-CoV-2 infection and subsequent COVID-19 lung disease. These results represent preclinical evidence for the use of such treatments in COVID-19 (99).

### RAAS system inhibitors

Angiotensin-converting enzyme inhibitors (ACEIs) and angiotensin II-1 receptor blockers (ARBs) are widely used in the treatment of arterial hypertension, heart failure and chronic kidney disease. Zhang et al. have confirmed that, the inpatient use of ACE inhibitor/ARB was associated with a lower all-cause mortality compared with non-users among hospitalized COVID-19 patients with hypertension (100).

### Inhibits cytokine storm

Cytokine storm is an important mechanism of COVID-19. Currently, the research drugs for COVID-19 are mainly focused on corticosteroids. A study involving 2,104 patients with COVID-19 showed that patients with invasive mechanical ventilation or oxygen therapy and without invasive mechanical ventilation had a significantly lower 28-day mortality after receiving glucocorticoids (6 mg/d, for up to 10 days) compared to the normal care group (101).

## Conclusions

As the COVID-19 pandemic persists and with the continued high incidence of cardiopulmonary complications in COVID-19 patients, we emphasize the key role that endothelial cells injury plays. By understanding its specific physiological and pathological mechanism and the current research into experimental clinical drugs, we believe that the rate of patient survival will be significantly improved. As many patients present with existing cardiovascular disease, care must be taken to perform a comprehensive evaluation paying particular attention to the risk of adverse drug interactions. More importantly, a review of previous pandemics has shown that thrombosis occurs whenever endothelial cell damage is present. The crucial role of PS in activating coagulation has led us to conclude that inhibiting PS externalization with early antithrombotic therapy can reduce mortality.

## Author contributions

LL prepared figures and wrote the manuscript. HJ, XW, and MX provided helpful comments and acquired data. JS and SW came up with the project, designed the study, contributed to successive drafts, and reviewed this manuscript. VN gave the revision advice and polished this review. All authors read and approved the final manuscript.

## Conflict of interest

The authors declare that the research was conducted in the absence of any commercial or financial relationships that could be construed as a potential conflict of interest.

## Publisher's note

All claims expressed in this article are solely those of the authors and do not necessarily represent those of their affiliated organizations, or those of the publisher, the editors and the reviewers. Any product that may be evaluated in this article, or claim that may be made by its manufacturer, is not guaranteed or endorsed by the publisher.
